# Temporal dynamics of intranasal oxytocin in human brain electrophysiology

**DOI:** 10.1093/cercor/bhab404

**Published:** 2022-01-04

**Authors:** Marie Zelenina, Maciej Kosilo, Janir da Cruz, Marília Antunes, Patrícia Figueiredo, Mitul A Mehta, Diana Prata

**Affiliations:** Instituto de Biofísica e Engenharia Biomédica, Faculdade de Ciências, Universidade de Lisboa, Lisboa 1749-016, Portugal; Section on Clinical and Computational Psychiatry, NIMH, NIH, MD 20814, USA; Instituto de Biofísica e Engenharia Biomédica, Faculdade de Ciências, Universidade de Lisboa, Lisboa 1749-016, Portugal; Laboratory of Psychophysics, Brain Mind Institute, École Polytechnique Fédérale de Lausanne (EPFL), Lausanne 1015 , Switzerland; Institute for Systems and Robotics–Lisbon (LARSyS) and Department of Bioengineering, Instituto Superior Técnico, Universidade de Lisboa, Lisboa 1049-001 , Portugal; Centro de Estatística e Aplicações e Departamento de Estatística e Investigação Operacional, Faculdade de Ciências, Universidade de Lisboa, Lisboa 1749-016, Portugal; Institute for Systems and Robotics–Lisbon (LARSyS) and Department of Bioengineering, Instituto Superior Técnico, Universidade de Lisboa, Lisboa 1049-001 , Portugal; INESC-ID, Instituto Superior Técnico, 1749-016 Lisboa, Portugal; Department of Neuroimaging, Institute of Psychiatry, Psychology and Neuroscience, King’s College London, London SE5 8AF, UK; Instituto de Biofísica e Engenharia Biomédica, Faculdade de Ciências, Universidade de Lisboa, Lisboa 1749-016, Portugal; Instituto Universitário de Lisboa (ISCTE-IUL), CIS-IUL, Lisboa 1649-026, Portugal; Department of Old Age Psychiatry, Institute of Psychiatry, Psychology and Neuroscience, King's College London, SE5 8AF London, UK

**Keywords:** electroencephalography, microstates, oxytocin, pharmacodynamics, resting-state

## Abstract

Oxytocin (OT) is a key modulator of human social cognition, popular in behavioral neuroscience. To adequately design and interpret intranasal OT (IN-OT) research, it is crucial to know for how long it affects human brain function once administered. However, this has been mostly deduced from peripheral body fluids studies, or uncommonly used dosages. We aimed to characterize IN-OT’s effects on human brain function using resting-state EEG microstates across a typical experimental session duration. Nineteen healthy males participated in a double-blind, placebo-controlled, within-subject, cross-over design of 24 IU of IN-OT in 12-min windows 15 min-to-1 h 42min after administration. We observed IN-OT effects on all microstates, across the observation span. During eyes-closed, IN-OT increased duration and contribution of A and contribution and occurrence of D, decreased duration and contribution of B and C; and increased transition probability C-to-B and C-to-D. In eyes-open, it increased A-to-C and A-to-D. As microstates A and D have been related to phonological auditory and attentional networks, respectively, we posit IN-OT may tune the brain for reception of external stimuli, particularly of social nature—tentatively supporting current neurocognitive hypotheses of OT. Moreover, we contrast our overall results against a comprehensive literature review of IN-OT time-course effects in the brain, highlighting comparability issues.

## Introduction

Intranasal oxytocin (IN-OT) has been used extensively to probe this neuropeptide’s role in human social cognition and behavior as we and others have reviewed (Evans et al. 2014; Leng and Ludwig 2016; Torres et al. 2018), and reported on (Neto et al. 2020). The potential therapeutic value of IN-OT has been studied in neuropsychiatric disorders with profound social and affective deficits, such as autism (Anagnostou et al. 2014), schizophrenia (De Berardis et al. 2013), and anxiety and depressive disorders (De Cagna et al. 2019)—with a general pattern of mixed findings, and lack of clear results (Bradley and Woolley 2017). IN-OT psychopharmacological studies need to collect outcome data within a time window that captures the pharmacodynamic effects of IN-OT. Generally, the timing of assessments can be determined by the plasma pharmacokinetics of a drug, or measurement of brain outcomes. We have summarized the timings used in previous studies in Table 1, also discussed them in detail in Box 1.

**Table 1:** Systematic summary of all previous human pharmacodynamics studies of intranasal oxytocin.

Study	N, gender	Design	Methods (OT extraction and quantification; or MRI technique)	IN-OT Dose	Post-administration time windows measured	Peak effect time window	Statistically significant effect time windows
**SALIVA**							
(Weisman et al. 2012)	10, m/f	Within-subj, double-blind PL	No extraction (lyophilization only for placebo); ELISA	24 IU	15 min, 30 min, 45 min, 1 h, 1 h20min, 1 h40min, 2 h, 3 h, 4 h	15 min	15 min, 30 min, 45 min,1 h, 1 h20min, 1 h40min, 2 h, 3 h, 4 h (all time windows)
(Huffmeijer et al. 2012)	57, f	Within-subj, double-blind PL	Extraction; ELISA	16 IU	Approx. 1 h15 min, approx. 2 h15 min	Approx. 1 h15 min	Approx. 1 h15 min, approx. 2 h15 min (all time windows)
(van Ijzendoorn et al. 2012)	47, f	Between-subj, double-blind PL	Extraction; ELISA	16/24 IU	1 h, 2 h, 3 h, 4 h, 5 h, 6 h, 7 h	1 h	1 h, 2 h, 3 h, 4 h, 5 h, 6 h, 7 h (all time windows)
(Daughters et al. 2015)	40, m	Within-subj, double-blind PL	No extraction (unclear if lyophilization); ELISA	24 IU	30 min, 1 h, 1 h30 min, 1 h45 min, 1 h48min	30 min	30 min, 1 h, 1 h30 min, 1 h45 min, 1 h48min (all time windows)
(Spengler et al. 2017)	116, m	Crossover, double-blind PL	Extraction; RIA	12/24/48 IU	12/48 IU: approx. 15 min, 40 min, 1 h20min, 1 h45min 24 IU: approx. 15 min, 45 min, 1 h25min, 1 h40min	NA	(all time windows)
**BLOOD**							
(Landgraf 1985)	3, m	No PL	Extraction; RIA	“﻿about 65 μ,” appr. 38 IU	10 min, 20 min, 30 min, 40 min, 50 min, 1 h, 1 h10min	NA	10 min, 20 min, 30 min
(Gossen et al. 2012)	8, m	No PL	Extraction; FEIA	26 IU	30 min, 1 h30 min, 2 h30 min, 3 h30 min	30 min	30 min, 1 h30 min (2 h30 min for some subj.)
(Striepens et al. 2013)	15, m	Between-subj, double-blind PL	Extraction; RIA	24 IU	15 min, 30 min, 45 min, 1 h, 1 h15 min, 1 h30 min	15 min	15 min, 30 min, 45 min, 1 h, 1 h15 min, 1 h30 min (all time windows)
(Quintana et al. 2015)^*^	16, m	Within-subj, double-blind, double-dummy, crossover	Extraction; ELISA	8/24 IU	10 min, 30 min, 1 h, 2 h	NA	10 min, 30 min, 1 h, 2 h (all time windows)
(Spengler et al. 2017)	116, m	Crossover, double-blind PL	Extraction; RIA	12/24/48 IU	24 IU: 45 min, 1 h25min, 1 h45min 12/48 IU: 1 h25min	NA	45 min, 1 h25min, 1 h45min (all time windows)
CSF							
(Striepens et al. 2013)	15, m	Between-subj, double-blind PL	Extraction; RIA	24 IU	45 min, 1 h, 1 h15 min	NA	only 1 h15 min
**MRI**							
(Paloyelis et al. 2016)	32, m	Between-subj design, single-blind PL	MRI: CBF	40 IU	25–38 min, 32—44 min, 39—51 min, 45—58 min, 52 min—1 h5min, 59 min—1 h11min, 1 h6min—1 h18min	39—51 min	39—51 min, 45—58 min, 52 min—1 h5 min
(Spengler et al. 2017)	116, m	Crossover, double- blind PL	MRI: BOLD	12/24/48 IU	24 IU: 15—38 min, 45 min—1 h8min, 1 h15 min—1 h38 min 12 IU: only 45 m–1 h8 48 IU: only 45 m–1 h8	NA	Only 45 min—1 h8min (only 24 IU, only in high-intensity fear condition)
(Martins et al. 2020b)	17, m	Crossover, double-blind PL	MRI: CBF	40 IU	15–23 min, 24—32 min, 35—43 min, 44—52 min, 1 h6min—1 h14min, 1 h15 min—1 h23min, 1 h27min—1 h35min, 1 h36min—1 h24 min	NA	15–23 min, 24–32 min, 35–43 min, 1 h27min—1 h35min

Approx. = approximately, BOLD = blood oxygenation level dependent, CBF = cerebral blood flow, CSF = cerebro-spinal fluid, RIA = radioimmunoassay, ELISA = enzyme linked immunosorbent assay, FEIA = fluorescent enzyme immunoassay; f = female, IU = international units, m = male, MRI = magnetic resonance imaging, NA = not available, PL = placebo, subj = subject.

Search used Google Scholar with keywords “oxytocin” AND “human” AND “time” AND (“mri” OR “eeg” OR “blood” OR “saliva” OR “csf”), complemented with a further backward and forward reference search

^*^This study used a breath powered closed-palate bi-directional device rather than the usual nasal spray.

Overall, studies demonstrate variability in the time windows tested and in the effects. Most of the findings used to inform subsequent decisions on psychopharmacological administration timings do not stem from brain activity measurements, but instead peripheral levels of OT in: saliva (Huffmeijer et al. 2012; van IJzendoorn et al. 2012; Weisman et al. 2012; Daughters et al. 2015), blood plasma (Gossen et al. 2012; Striepens et al. 2013), and cerebrospinal fluid (CSF) (Striepens et al. 2013). Out of 11, only three studies have used magnetic resonance imaging (MRI) to examine the effects of IN-OT on the human brain across time, two measuring cerebral blood flow (CBF) (Paloyelis et al. 2016; Martins et al. 2020b), and one using blood oxygenation-level-dependent imaging (BOLD) (Spengler et al. 2017). As such, findings that seem consistent (i.e., because they overlap in time) may not be in fact directly comparable given the different tissues  in

**Box 1**

Systematic overview of previous findings addressing temporal pharmacodynamics of IN-OT in humans.

**Saliva studies**

Out of the five saliva studies (which given the OT administration was intranasal, warrant healthy skepticism—see below) three used 24 IU of IN-OT (Weisman et al. 2012; Daughters et al. 2015), one used 16 IU (Huffmeijer et al. 2012), and two compared the effect of different dosages: 24 IU versus 16 IU (van IJzendoorn et al. 2012), 24 IU versus 12 IU versus 48 IU (Spengler et al. 2017). The time span of measurements ranged from 15 min (Weisman et al. 2012; Spengler et al. 2017) to 7 h post-administration (van IJzendoorn et al. 2012). In all studies, saliva OT levels were significantly elevated during the whole observation period. The peak of the effect varied between saliva studies but was always at the earliest measurement point in all five studies, at 15 min (Weisman et al. 2012; Spengler et al. 2017), at 30 min (Daughters et al. 2015), at 1 h(van IJzendoorn et al. 2012), and at 1 h 15 min (Huffmeijer et al. 2012). However, caution interpreting the detected effects of IN-OT is needed, since the OT levels measured in the saliva may be partially originating from the nasal cavity to the back of the throat, rather than a consequence of the effect of OT on the brain (Daughters et al. 2015; Quintana et al. 2018). This argument is the main limitation of the saliva method—which seems difficult to control and thus we recommend that IN-OT studies refrain from measuring OT in saliva. Another limitation is the high rate of non-specific binding between non-OT compounds to OT antibodies ﻿in the preferred commercial saliva ELISA (enzyme linked immunosorbent assay) kit, which can lead to artificially elevated concentrations of OT in the sample (McCullough et al. 2013). The last issue was addressed in only two studies by using an improved (Daughters et al. 2015) or a different (Spengler et al. 2017) kit. Furthermore, three of the saliva studies (Huffmeijer et al. 2012; van IJzendoorn et al. 2012; Weisman et al. 2012) included female participants, and only van IJzendoorn et al. accounted for the menstrual phase. One of those (Weisman et al. 2012) mixed male and female participants and include sex in the analysis model. We argue that both sex and menstrual cycle phase should be taken into account because of is evidence that OT baseline levels are 3x higher in women than in men, as measured in plasma (Marazziti et al. 2019), that menstrual cycle impacts OT levels (Mitchell et al. 1981; Stock et al. 1991; Salonia et al. 2005), as well as several reports on sex-related differences in functional effects of OT (Evans et al. 2014).

**Blood plasma studies**

Three studies measured OT concentration in blood plasma (Gossen et al. 2012; Striepens et al. 2013; Spengler et al. 2017). All used only male participants and 24 IU (Striepens et al. 2013), 26 IU (Gossen et al. 2012), or 12/24/48 IU (Spengler et al. 2017) of IN-OT. In agreement with saliva studies, plasma studies have showed peak effects at the earliest measurement point of 15 min (Striepens et al. 2013) and 30 min (Gossen et al. 2012) post-administration. One study did not report the peak time window (Spengler et al. 2017). As for the duration of the effect, two studies observed significant differences from baseline at the latest measurement point: at 1 h45min and 1 h30 min, respectively (Striepens et al. 2013; Spengler et al. 2017). Gossen et al. had OT concentrations return to baseline levels at 1 h30 min for some participants and at 2 h30 min for all participants, even when the last measurement was at 3 h (Gossen et al. 2012). This highlights the previously noted (Quintana et al. 2018) tissue-dependent nature of OT pharmacodynamics, since saliva studies showed, instead, a significant effect up to 7 h post-administration. There was also another study (Quintana et al. 2015), which found a significant increase of OT in blood plasma at 10, 30, 60, and 120 min post-administration with 8 IU as well as with 24 IU (using a novel Breath Powered device, rather than the usual nasal spray).

Blood plasma studies have similar methodological drawbacks as saliva studies in terms of the inference of IN-OT’s temporal dynamics in the brain. Nevertheless, even though plasma OT concentration does not necessarily reflect basal OT levels in the brain (McCullough et al. 2013; Leng and Ludwig 2016; Martins et al. 2020a), these have shown positive correlation after IN-OT and after stressor state measures (Valstad et al. 2017) and in some measurement methods (Lefevre et al. 2017). Second, some studies used non-specific antibodies, which may make the measurements less sensitive to the drug (Haraya et al. 2017). Third, some studies (Weisman et al. 2012; Daughters et al. 2015) did not use the OT extraction step prior to its quantification via enzyme immunoassay (EIA), which some authors suspect may render results invalid as extra immunoreactive products with similar structure to OT may also be inadvertently measured along with OT (McCullough et al. 2013). Commercially available EIA assays without extraction output values two orders of magnitude higher than those obtained using conventional RIA methods with extraction; and these extra immunoreactive products’ levels do vary across individuals, and physiological states (McCullough et al. 2013). (Lyophilization has sometimes been used as a replacement of the extraction step in the ELISA—this may have been the case of one study (Daughters et al. 2015) although the references they used cast doubt; and was the case of another (Weisman et al. 2012) but the use was only in placebo samples, which could be an important confounding factor for their reported effects of drug.) Adding to this, two of the three studies used a small number of participants (Gossen et al. 2012; Striepens et al. 2013). Finally, authors pointed out (Gossen et al. 2012), plasma measurements are less indicative of OT availability in brain than CSF measurements (Mens et al. 1983) and thus IN-OT administration may affect neural function even after OT plasma levels have returned to baseline. To address this issue, one of those three studies (Striepens et al. 2013) added a measurement of OT in the CSF, to be compared with that in plasma, at half of the time windows (see below).

**Cerebrospinal fluid studies**

OT concentrations in the CSF were significantly increased only at 1 h15 min post-administration, i.e., the latest measurement point (Striepens et al. 2013). This is in contrast to OT plasma levels, which were elevated from 15 min to 1 h45 min. Also, there was no correlation between concentrations of OT in plasma and CSF samples, which raises an additional concern when it comes to comparing blood and brain findings; although a later study found levels of endogenous plasma OT to significantly and positively predict endogenous OT levels in CSF (Carson et al. 2015). One limitation of Striepens et al. (2013)‘s study was that due to the underlying invasive and complicated procedure, the CSF measurements could not be taken earlier than 45 min post-administration; however, as the authors state (Striepens et al. 2013), it is unlikely OT levels in CSF had risen and fallen before thin time window. A methodological explanation the authors provide for the late onset of the OT increase in the CSF, compared to blood or saliva, is that the CSF measurement is taken via a lumbar puncture, which is down the spinal cord, and it might have taken that amount of time for OT, either exogenous (IN-OT) or exogenously stimulated (hypothalamically released due to stimulation by intranasal administration), to travel down the spinal cord (Striepens et al. 2013). In that case, an effect of IN-OT in the brain could have been achieved earlier.

**Magnetic resonance imaging studies**

The three studies that we are aware of which measured the functional influence of IN-OT administration on the human brain directly and across time (Paloyelis et al. 2016; Spengler et al. 2017; Martins et al. 2020b), all used MRI. Spengler et al. (Spengler et al. 2017) administered 24 IU and tracked the BOLD contrast in response to fearful faces images, from 15 min to 100 min post-administration. As expected from previous studies (Gamer et al. 2010), they observed the expected left amygdala inhibition response to fear to be most effective at the 45–70 min interval post-administration. This pattern was not observed with either lower (12 IU) or higher doses (48 IU), the latter dose showing in fact an opposite trend. This suggests a possible reversal of the functional effect of IN-OT for this higher dose and calls for extra care when interpreting studies that used different OT doses. This is in agreement with the inverted U-shaped dose-effect dependency of OT, whereby the observable effects of IN-OT diminish with high doses, which was put forward based on diverse evidence at both the neurophysiological (Wynn et al. 2019) and behavioral (Zhong et al. 2012); (Borland et al. 2019) levels. Overall, while this study adds an important insight on the temporal and dose effect of IN-OT, its focus on the amygdala leaves the question of IN-OT’s effects on the rest of the brain open.

Another study (Paloyelis et al. 2016) recorded changes in CBF across the whole brain in a resting-state functional MRI (fMRI) session, after 40 IU of IN-OT/PL administration, and applied pattern recognition to predict probabilities of the nasal spray effect. After comparing the predicted to the administrated nasal spray effect, they observed 80% classification accuracies in the IN-OT group across the whole post-administration period (25–78 min), which was significantly different from chance. In the group, they noticed 38%–81% accuracies, which was not different from chance except, surprisingly, for 32 to 44 min post-administration. They also localized the IN-OT across the whole post-administration period with univariate analysis and found significant results in four clusters spanning the midbrain, basal ganglia, limbic system, and cingulate, frontal, parietal temporal cortices and the cerebellum. This study (Paloyelis et al. 2016) provides an important insight into the effect on IN-OT at the whole brain level, but given the results of Gamer et al. (2010) (Gamer et al. 2010) it is not clear whether this result is representative of the lower and most commonly used (for research) dose of 24 IU or limited to the higher dose where the effect was not so clear (40 IU). Finally, although the authors found a significant difference in CBF between the IN-OT and placebo groups at the pre-administration baseline, the classification accuracies in the IN-OT and in the groups were compared against their respective baselines.

Recently, the same research group (Martins et al. 2020b) compared the CBF resulting from the usual intranasal nose spray, intranasal nebulizer, and intravenous administration of, again, 40 IU of OT, in a time span from 15 min to 1 h24min post-administration. They report a significant effect of IN-OT, or a significant interaction between treatment and time, on separate brain clusters, in time windows 15–23 min, 24–32 min, 35–43 min, and 1 h27min—1 h35min post-administration—thus with a hiatus of 44 min to 1 h27min where no effect was detected. Results of Paloyelis et al. (2016) and Martins et al. (2020b) are not fully comparable or consistent (the first reports no effect from 25 to 44 min, it starting from then on to 1 h05min,while the later study showed a non-overlapping effect that starts as early as 15 min and last only up to 43 min), even though a similar experiment design and measure (CBF), experimental site, and a matching IN-OT dose (40 IU) was used. The second effect detected in the later study (starting as late as 1 h27 min) was in a timing not tested in the former study, so the replication would have not been possible. Location wise, both studies found largely overlapping and widespread regional effect, although only in the later study could they directly be related to the timeline results, since the former reports the analyses of time and location in separate, and different types of, models.

which OT has been measured. Plasma and saliva OT measurements seem not to represent well those in the brain, especially at basal or trait level (McCullough et al. 2013; Leng and Ludwig 2016; Martins et al. 2020a), even though there is evidence that plasma OT is positively associated with CSF OT: after IN-OT administration, after an experimental stressor (via meta-analysis (Valstad et al. 2017)), and when measurement methodology is carefully considered (Lefevre et al. 2017). Other limitations stemming from the current study pool include: 1) limited amount of subjects and lack of gender control; 2) non-specific binding agents in OT quantification in saliva and blood; 3) use of a range of OT doses between studies, whose effects cannot thus be inter-extrapolatable given evidence of an inverted-U shaped dose-effect relationship (Wynn et al. 2019); and 4) the problematic use of unextracted samples in fluid studies (McCullough et al. 2013).

Surprisingly, given its ability to record time-varying changes in neural activity (traceable to post-synaptic potentials of the pyramidal neurons (Kirschstein and Köhling 2009)) electroencephalography (EEG) has not yet been used to track pharmacodynamics of IN-OT; i.e., IN-OT induced changes in brain activity at different times following drug administration. This lack is even more relevant for the design and interpretation of IN-OT EEG studies, which have been steeply accumulating (Perry et al. 2010; Mu et al. 2016; Singh et al. 2016; de Bruijn et al. 2017; Luo et al. 2017; Rutherford et al. 2018; Schiller et al. 2019), and have assessed, for example, its effect on EGG time-frequency changes during videos of biological motion (Perry et al. 2010) and images of sadistic context (Luo et al. 2017). These studies have mostly used 24 IUs, and healthy, predominantly male participants, with exceptions (Singh et al. 2016; Rutherford et al. 2018), and two used a resting-state paradigm (Rutherford et al. 2018; Schiller et al. 2019). For example, cross-frequency coupling between slow and fast waves has been found to be decreased under IN-OT, suggesting it may modulate integration of motivational and cognitive processes (Rutherford et al. 2018). This effect was apparent even at the latest point amongst EEG studies (70 min after drug administration); which mostly started at 40 min (Mu et al. 2016; de Bruijn et al. 2017; Luo et al. 2017) or 45 min (Perry et al. 2010; Singh et al. 2016; Schiller et al. 2019).

A novel and promising EEG analysis approach is based on the observation that the scalp voltage distribution, although dynamically changing over time, displays a limited number of characteristic topography patterns, so-called “microstates” (Lehmann et al. 1987). Four canonical microstate classes (labeled A, B, C, and D) have been identified and consistently reported across studies (Michel and Koenig 2018). Each microstate remains in a quasi-stable state for 60–120 ms and then rapidly transitions to a different microstate. Although the discreetness of individual microstates has also been questioned (Mishra et al. 2020), this approach has an important advantage over other EEG measures. First, since signals are simultaneously considered from across the entire scalp, large-scale brain networks can be assessed. Second, the four canonical resting-state microstates seem to correspond to well-known fMRI resting-state networks (Britz et al. 2010; Musso et al. 2010; Yuan et al. 2012; Milz et al. 2016; Custo et al. 2017). We have recently confirmed this by showing microstates to predict fMRI dynamic functional connectivity states with 90% accuracy, using simultaneous EEG and fMRI recording (Abreu et al. 2021). Furthermore, we have also highlighted the utility of microstates in identifying potential endophenotypes of psychiatric disorders, such as schizophrenia (da Cruz et al. 2020), for which OT has been proposed by others as a potential intervention to alleviate social impairments (De Berardis et al. 2013; Shilling and Feifel 2016). In sum, a microstates approach can be revealing of broad effects of IN-OT across the brain, and across post-administration time, with the potential of to bridge resting state findings across neuroimaging techniques, as well as to inform its therapeutic use. Despite that, so far only one resting-state EEG study (Schiller et al. 2019) measured the effect of IN-OT on human resting-state EEG using a microstates approach. They showed that IN-OT significantly increased duration of all microstates, decreased occurrence of microstates B and C, increased the contribution of microstate D, and decreased transitions from microstate B to C (see section Materials and Methods, for microstates features’ definitions). Nevertheless, no conclusions regarding the time course of IN-OT effects on the brain can be derived from this study, as it uses only a single, 5 min-long (40–45 min post-administration), recording time window.

In the present study, we aimed to characterize the temporal dynamics of IN-OT using microstate analysis of EEG data. We tracked IN-OT-induced changes across the whole brain, and across almost 2 h of experimental time, on microstates function during eyes-open and eyes-closed resting-state. We used 24 IU with a throughout double-blind randomized placebo-controlled cross-over design (in 6 time windows: 15–27, 30–42, 45–57, 60–72, 75–87, and 90–102 min, post-administration), in a young male sample, at the same time of day. The use of a dose commonly employed in neuroscience research brings the advantage of aiding interpretation of previous both positive and negative behavioral and brain phenotype findings. We collected data from both eyes open (EO) and eyes closed (EC) conditions, as there is evidence of different microstate patterns between the conditions (Seitzman et al. 2017), as well as across-scalp means of EEG frequency waves and their topographies (Barry et al. 2007), and because IN-OT effects could not be assumed to be the same across eye statuses. In addition, we briefly discuss the role of OT on the specific microstates in relation to the cognitive network function they may reflect. We hope our findings can inform comparability between past studies, and the design of future IN-OT studies, especially those aiming to employ psychological tasks and thus naturally concerned with the start, duration, and end of task presentation and psychophysiological recording times; as well as to entice hypothesis-generating reflections on the role of OT in human cognition.

## Materials and Methods

### Participants

We recruited 20 young healthy, male, Portuguese adults, one of which was excluded due to EEG recording problems, through mailouts and pamphlets in the university community and online social networks. The participants used for analysis (*n* = 19) were on average 27 years old (mean = 27.1, SD = 4.02, range = 18–35 y). We applied the following exclusion criteria: history of endocrinological, cardiovascular, or neurological disorders, substance abuse, blocked nose; use of cannabis 2 weeks prior, and alcohol consumption, drugs or medication 24 h prior, and smoking 2 h prior to the experimental session; caffeine consumption or heavy physical exercise or sexual activity on that day. Screening for exclusion and inclusion criteria was performed via self-report during an initial phone interview and in person, immediately prior to the recording. All participants gave their written informed consent, received financial compensation for their time. The study was approved by the Ethics Committee of the Lisbon Medical Academic Center (Centro Académico Médico de Lisboa, CAML) and complies with national and EU legislation for clinical research.

### Experimental Procedure

The experimental session took place at a quiet room of the CAML’s Clinical Research Centre (Centro para Investigação Clínica) in the Hospital de Santa Maria, Lisbon, Portugal. We used a double-blind (throughout data collection up to statistical analysis, inclusive), randomized placebo-controlled, cross-over design, whereby each participant took part in two sessions: one for IN-OT and once for placebo administration, in a counterbalanced order. The IN-OT administration of 24 IU was via 3 puffs of 0.1 mL each, in each nostril, from a 40 IU 5 mL Syntocinon bottle (using the Novartis formula—batch H5148 produced by Huningue Production, France) or an identical placebo bottle (with the same ingredients, except OT—batch 170317.01 produced by VolksApotheke Schffhausen, Switzerland), both supplied by Victoria Apotheke Zürich, Switzerland. Drug acquisition, storage, and randomization of drug administration was performed and controlled by the hospital’s pharmacy. IN-OT/placebo administration was at 2:22 pm (SD = 29 min) for all participants, and EEG recording started 12 min prior to the drug administration, to restrict the impact of the circadian rhythm on baseline endogenous OT levels (Perlow et al. 1982; Reppert et al. 1984; Devarajan and Rusak 2004). Upon arrival, participants confirmed all eligibility criteria via questionnaire and their health state was assessed via medical examination, which included heart rate, blood pressure, and electrocardiogram measurements. In the second session, only the eligibility questionnaire was administered. OT and placebo sessions were seven days apart (mean = 7.3, SD = 1.95 days).

During the 7 resting-state EEG recording windows (−12–0 min pre-administration and 15–27, 30–42, 45–57 min, 1–1 h 12min, 1 h 15 min–1 h 27min, 1 h 30 min–1 h 42min after administration), participants were instructed to stay still, not engage in any cognitive process, and to practice meditation or mind-wandering (more details in Supplementary Figure S1). Each recording window (“time window”) included 5-min recording with eyes closed, followed by 5-min recording with eyes open. Participants compliance with eye-status instructions was confirmed via an eye tracker (EyeLink 1000 plus, SR Research, Canada). At the end of each time window, participants filled three Likert scales: for alertness (1—alert, 5—sleepy), excitation (1—excited, 5—calm), and desire to socialize (1—desire to socialize, 5—desire to be left alone).

### E‌EG Data Acquisition and Processing

We recorded the EEG signal at a 1000 Hz sampling rate using a 64-channel Brain Vision actiCHamp system (Brain products, München, Germany). Electrodes were placed according to the 10/20 system, with the ground electrode at the central front location on the cap. To ensure optimal signal quality, we aimed to calibrate all electrodes below 5 kΩ, and kept all electrodes below 44 kΩ for all participants. During recording, electrodes on the left and right mastoid served as a reference. We placed horizontal electrooculogram and vertical electrooculogram electrodes to record eye-movements and blinks; however, due to excessive noise, these data were discarded and ocular artifacts were removed using Independent Component Analysis (ICA; see next section).

Offline EEG data were preprocessed in Matlab, version R2019b, using an automatic pre-processing pipeline (APP; da Cruz et al. 2018) based on EEGLAB toolbox (Delorme and Makeig 2004) v12. We designed this pipeline specifically for, and tested it on, resting-state data; and it was shown to perform similarly to supervised pre-processing done by EEG experts, and to outperform existing alternatives (Bigdely-Shamlo et al. 2015; Hatz et al. 2015) in terms of the amount of data lost. The pre-processing consisted of the following steps: 1 to 30 Hz bandpass filtering, powerline noise removal, down-sampling to 250 Hz, re-referencing to the bi-weight estimate of the mean of all channels, removal and 3D spline interpolation of bad channels; removal of bad epochs; independent component analysis (ICA) to remove eye movement, muscular and bad channel related artifacts; re-referencing to common average reference. After automatic pre-processing, data were visually inspected for any additional artifacts, which were removed if needed. We decided, posterior to data collection and preprocessing but prior to data analysis, to limit our analysis to the first artifact-free 176 s, of each condition, in each time window, in order to: 1) minimize effects of drowsiness or sleepiness (which may take place after 3 min of non-stop recording (Tagliazucchi and Laufs 2014)), and 2) to be directly comparable to the (only) previous IN-OT EEG microstates study (Schiller et al. 2019), which used these data average length.

### Microstate Extraction and Quantification

We extracted microstates of each time window and each condition (eyes-closed/eyes-open) separately, using the Microstates toolbox (Koenig 2017) for EEGLAB. First, we further bandpass-filtered the data from 2 to 20 Hz, as commonly done in the literature (Koenig et al. 2002; Lehmann et al. 2005; Pascual-Marqui et al. 2014; Milz et al. 2016), including in the only existing study that employed microstates in IN-OT research (Schiller et al. 2019). Next, for each of the seven time windows, we concatenated each subject’s epochs into one and calculated the Global Field Power (GFP) across all electrodes over this observation period (that equaled 176 s), for eyes closed and open separately. GFP is calculated as the standard deviation across electrodes at a given time window and it quantifies the overall potential strength across the given set of electrodes. Local maxima of GFP represent the highest signal-to-noise ratio, and are associated with a stable EEG topography (Lehmann et al. 1987).

Next, in moments of time that corresponded to local GFP maxima in each time window, we extracted scalp topographies and clustered them into four classes, A, B, C, and D (following the previous literature, which converged on four optimal microstate classes; see 37,52,53), using the atomize-agglomerate hierarchical clustering algorithm (AAHC; (Murray et al. 2008; Tagliazucchi and Laufs 2014)). The clustering was done on an individual level, and then across all participants in both recording sessions (but separately for each time window). Next, we assigned the microstate labels A, B, C, and D to the grand-grand average of both sessions based on visual inspection of the grand-grand average maps, and on their similarity with the 4 canonical microstates found in the literature (Michel and Koenig 2018), and back-propagated the sorting to the individual maps (i.e., participants’ topographies at the GFP peaks were labeled as microstates A, B, C, and D based on spatial correlation with the grand-grand average). In both sessions, the similar (and thus comparable) four microstates resembled the four class model maps consistently identified in the literature (Koenig et al. 2002); (Michel and Koenig 2018)—see Supplementary Figure S2. Using spatial correlation, we confirmed that the microstates in IN-OT and placebo sessions were (see Supplementary Material B for detailed analysis and results). The spatiotemporal dynamics of the four microstates categories were quantified as standard (Michel and Koenig 2018) by measuring their: 1) duration (s), i.e., the mean time the microstate was present; 2) occurrence (times/s); i.e., the mean frequency it was present; (3) contribution (%); i.e., the proportion of total time it was present; and (4) delta transition probability from one microstate to another (%); i.e., the relative amount of times microstate X was followed by microstate Y, calculated as the difference between the observed and the predicted value given previously observed occurrences.

### Statistical Analysis

We designed a series of linear mixed models (LMM) using R (*lme4* package and the *lmer* function (Bates et al. 2015)); see Supplementary Material C for syntax and full results). These models allow for the presence of missing data, as well as to specify drug-varying covariates (such as the baseline values, which differed in each drug session per participant), and take into account inter-individual random differences. Models were run separately for each microstate feature as dependent variable (duration, occurrence, contribution) of each microstate (A-D), and for microstate-to-microstate transition probability (in 12 models, i.e., one per every possible transition pair). Each model estimated the fixed effects of the categorical factors Drug session (IN-OT, placebo) and Time (post-administration time windows: 1, 2, 3, 4, 5, 6), and their interaction. For baseline correction, the microstate feature at time 0 (i.e., before administration) in the placebo and the IN-OT session was included as a covariate of no interest in each model. We used an identical LLM to analyze behavioral scales (one model per scale). The number of degrees of freedom, and consequently *P*-values, were calculated using Type III analysis of variance with the Satterthwaite’s method. If main effects of drug and/or interactions between drug and time were statistically significant (uncorrected *P-value* < 0.05), we performed pairwise comparisons on estimated marginal means (degrees of freedom estimated using the Kenward–Roger method) using EMMEANS package for R. The main effects of time were not interpreted, as it was not relevant to our hypothesis—but is reported as Supplementary Material C. Finally, although the drug inhalation procedure was standardized, we further ensured that there was no confounding effect due to differences in the amount of inhaled IN-OT (details in Supplementary Material B).

## Results

**Figure 1:**
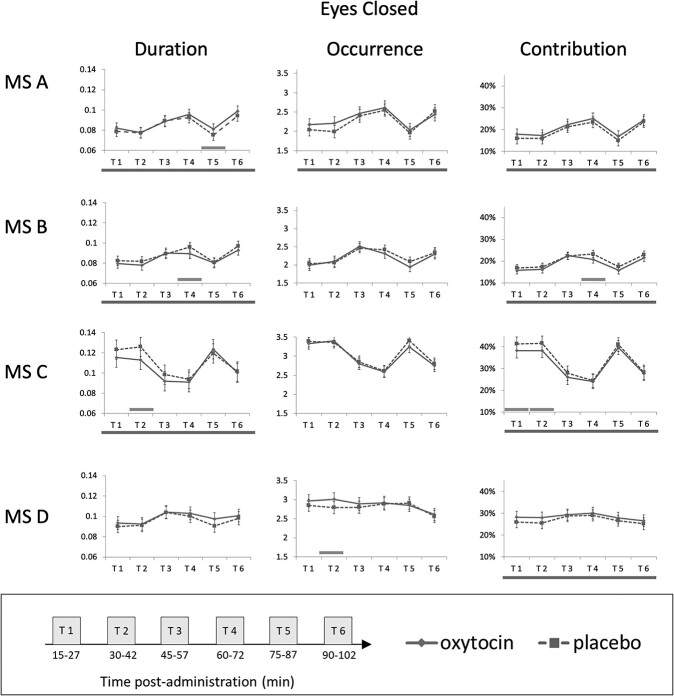
IN-OT- and PL-induced changes in the microstate features for the eyes-closed condition over the post-administration observation period. Dashed line corresponds to placebo and continuous line to oxytocin. For each subplot, bottom line across all time windows signals a main effect of drug. Significant pairwise comparisons (oxytocin vs. placebo) at specific time windows are signaled with a green bar above the *x* axis. Error bars: 95% Cl. Time windows: 1 = 15—27 min after drug (IN-OT/PL) administration, 2 = 30–42 min, 3 = 45–57 min, 4 = 1 h–1 h12min, 5 = 1 h15 min–1 h27min, 6 = 1 h30 min–1 h42 min. MS = microstate.

**Figure 2:**
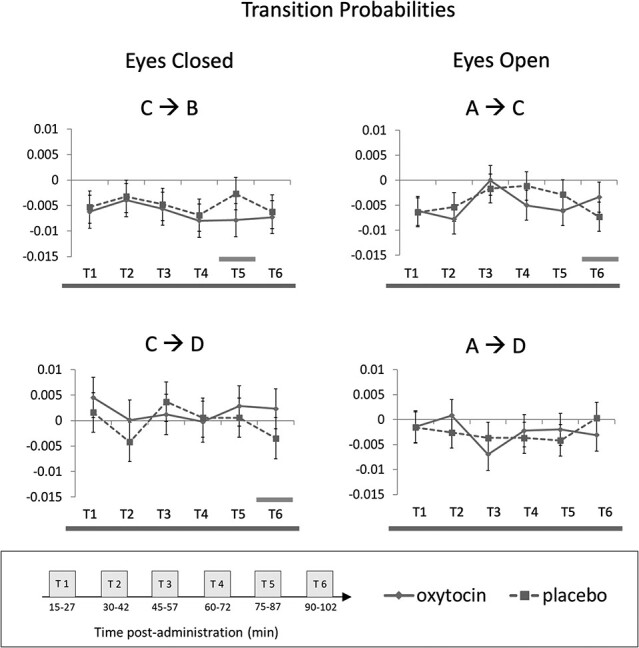
IN-OT- and PL-induced changes in transition probabilities for eyes-closed and eyes-closed recording, where main effects of drug or drug × time interaction was detected. Dashed line corresponds to placebo and continuous line to oxytocin. For each subplot, bottom line across all time windows signals a main effect of drug or interaction. Significant pairwise comparisons (oxytocin vs. placebo) at specific time windows are signaled with a bar above the *x* axis. Error bars: 95% Cl. Time windows: 1 = 15—27 min after drug (IN-OT/PL) administration, 2 = 30–42 min, 3 = 45–57 min, 4 = 1 h–1 h12min, 5 = 1 h15 min–1 h27min, 6 = 1 h30 min–1 h42 min. MS = microstate.

All main effects and interactions, for both the eyes-closed and eyes-open conditions, for each microstate feature and for each microstate (A, B, C, and D), are reported in Supplementary Material C and summarized in Table 2. For eyes-closed, the results for contribution, duration, and occurrence are plotted in Figure 1, and for both eyes-closed and eyes open status, transition probabilities are plotted in Figure 2 (no other effects being statistically significant in this condition). Below, we describe all statistically significant effects (or notable trends) of IN-OT (main or interactions), there being none on mood scales.

**Table 2:** Summary of selected microstate features and transition probabilities’ main effects of drug (intranasal oxytocin vs. placebo), or interaction between drug and time, and the corresponding pairwise comparisons between IN-OT and placebo in time windows where the comparison was significant (*P* < 0.05, with the two borderline-significant trends also described). Time windows: 1 = 15–27 min after drug (IN-OT/PL) administration, 2 = 30–42 min, 3 = 45–57 min, 4 = 1 h–1 h12min, 5 = 1 h15 min–1 h27min, 6 = 1 h30 min–1 h42 min; MS = microstate. D = drug, T = time, IN-OT = intranasal oxytocin

Microstate label	Microstate measure	IN-OT effects	Time windows (pairwise comparisons)	Direction
**Closed eyes**				
MS A	Duration	D: *F*(1, 190) = 5.05, *P* = 0.026	T5: *t*(189) = 1.98, *P* = 0.049	IN-OT ↑
	Occurrence	n.s. [*F*(1, 194) = 3.83, *P* = 0.052]	n.s.	IN-OT ↑
	Contribution	D: *F*(1, 190) = 8.57, *P* = 0.004	n.s.	IN-OT ↑
MS B	Duration	D: *F*(1, 194) = 5.82, *P* = 0.017	T4: *t*(190) = −2.31, *P* = 0.022	IN-OT ↓
	Occurrence	n.s.	n.s.	-
	Contribution	D: *F*(1, 193) = 10, *P* = 0.002	T4: *t*(190) = −2.49, *P* = 0.013	IN-OT ↓
MS C	Duration	D: *F*(1, 188) = 6.68, *P* = 0.011	T2: *t*(188) = −3.04, *P* = 0.003	IN-OT ↓
	Occurrence	n.s.	n.s.	-
	Contribution	*F*(1, 189) = 10.59, *P* = 0.001	T1: *t*(188) = −2.34, *P* = 0.020 T2: *t*(188) = −2.53, *P* = 0.012	IN-OT ↓
MS D	Duration	n.s. [D: *F*(1, 191) = 3.60, *P* = 0.059]	n.s.	IN-OT ↑
	Occurrence	D: *F*(1, 186) = 4.36, *P* = 0.038	T2: *t*(188) = 2.52, *P* = 0.013	IN-OT ↑
	Contribution	D: *F*(1, 191) = 5.65, *P* = 0.018	n.s.	IN-OT ↑
Transitions	C → B	D: *F*(1, 190) = 4.58, *P* = 0.034	T5: *t*(189) = −2.82, *P* = 0.005	IN-OT ↓
	C → D	D: *F*(1, 191) = 4.06, *P* = 0.045	T6: *t*(189) = 2.34, *P* = 0.021	IN-OT ↑
**Open eyes**				
Transitions	A → C	D^*^T: *F*(5, 188) = 2.95, *P* = 0.014	T6: *t*(189) = 2.34, *P* = 0.021	IN-OT ↑
	A → D	D^*^T: *F*(5, 188) = 2.30, *P* = 0.046	n.s.	N/A

### Eyes-Closed Data

#### Microstate A

Duration of microstate A was longer in the IN-OT (*M* = 0.087, SE = 0.002) than the placebo session (*M* = 0.085, SE = 0.002; *F*(1, 190.4) = 5.05, *P* = 0.026) irrespective of time. Pairwise comparisons on estimated marginal means indicated that microstate A duration was longer for IN-OT than placebo, particularly in time window 5 (*t*(189) = 1.98, *P* = 0.049). The occurrence of microstate A appeared higher under IN-OT (*M* = 2.320, SE = 0.061) compared to placebo (*M* = 2.250, SE = 0.060), irrespective of time, but this difference was not statistically significant (F(1, 194) = 3.83, *P* = 0.052). Contribution of microstate A was larger for IN-OT (*M* = 0.21%, SE = 1%) than placebo (*M* = 19%, SE = 1%; *F*(1, 190) = 8.57, *P* = 0.004). Pairwise comparisons between IN-OT and placebo at separate time windows did not reveal significant effects.

#### Microstate B

Duration of microstate B was shorter under IN-OT (*M* = 0.085, SE = 0.002) than placebo (*M* = 0.088, SE = 0.001; F(1, 194.3) = 5.82, *P* = 0.017) irrespective of time. Duration was shorter under IN=OT than placebo particularly in time window 4 as revealed by pairwise comparisons (1–1 h12min post-administration; *t*(190) = −2.31, *P* = 0.022). Contribution of microstate B was smaller in IN-OT (*M* = 19%, SE = 1%) than placebo (*M* = 20%, SE = 1%; *F*(1, 193.5) = 10, *P* = 0.002) also particularly in time window 4 (1 h–1 12 min; *t*(190) = −2.49, *P* = 0.013).

#### Microstate C

Duration of microstate C was shorter under IN-OT (*M* = 0.106, SE = 0.004) than placebo (*M* = 0.110, SE = 0.004; *F*(1, 188) = 6.68, *P* = 0.011), particularly in time window 2 (30–42 min; *t*(188) = −3.04, *P* = 0.003). Contribution of microstate C was smaller in the IN-OT (*M* = 32%, SE = 1%) session than placebo (*M* = 34%, SE = 1%; *F*(1, 189) = 10.59, *P* = 0.001), particularly in time windows 1 (15–27 min; *t*(188) = −2.34, *P* = 0.0203) and 2 (30–42 min; *t*(188) = −2.53, *P* = 0.012).

#### Microstate D

The duration of microstate D was longer for IN-OT (*M* = 0.099, SE = 0.002) than placebo (*M* = 0.096, SE = 0.002), irrespective of time, but this did not reach statistical significance (*F*(1, 191.3) = 3.6, *P* = 0.059). Occurrence of microstate D was increased in IN-OT session (*M* = 2.88, SE = 0.059) compared to placebo (*M* = 2.8, SE = 0.059; *F*(1, 186) = 4.36, *P* = 0.038), particularly in time window 2 (30–42 min; *t*(188) = 2.52, *P* = 0.013). Contribution of microstate D was larger for IN-OT (*M* = 28%, SE = 1%) than placebo (*M* = 27%, SE = 1%; *F*(1, 191.2) = 5.65, *P* = 0.018); pairwise comparisons did not point to significant time windows.

#### Transition Probabilities

Transition C → B was decreased for the IN-OT (*M* = − 0.006, SE = 0.001) compared to placebo (*M* = − 0.005, SE = 0.001; *F*(1, 189.85) = 4.58, *P* = 0.034), particularly in time window 5 (1 h15 min—1 h27 min; *t*(189) = −2.82, *P* = 0.005). Transition C → D was increased for IN-OT (*M* = 0.002, SE = 0.001) compared to placebo (*M* = − 0.0002, SE = 0.001; *F*(1, 191.18) = 4.063, *P* = 0.045), particularly in time window 6 (1 h30 min—1 h42 min; *t*(189) = 2.34, *P* = 0.021).

### Eyes-Open

#### Transition Probabilities

Transition A → C showed a drug by time interaction (*F*(5, 188) = 2.95, *P* = 0.014), such that IN-OT elicited increased transition probability (*M* = − 0.003, SE = 0.002) relative to placebo (*M* = − 0.007, SE = 0.001), in time window 6 (1 h30 min—1 h42 min; *t*(192) = 2.15, *P* = 0.033) whilst the opposite (non-significant) trend was seen in time windows 2, 4, and 5, from inspection of Figure 2. Transition A → D also showed a drug by time interaction (*F*(5, 187.76) = 2.30, *P* = 0.046). Drug effect was not significant at any individual time window, but, from inspection of Figure 2, time window 5 is the only showing a trend, whereby IN-OT shows a greater transition probability (*M* = − 0.002, SE = 0.002) vs. placebo (*M* = − 0.004, SE = 0.002).

## Discussion

**Figure 3:**
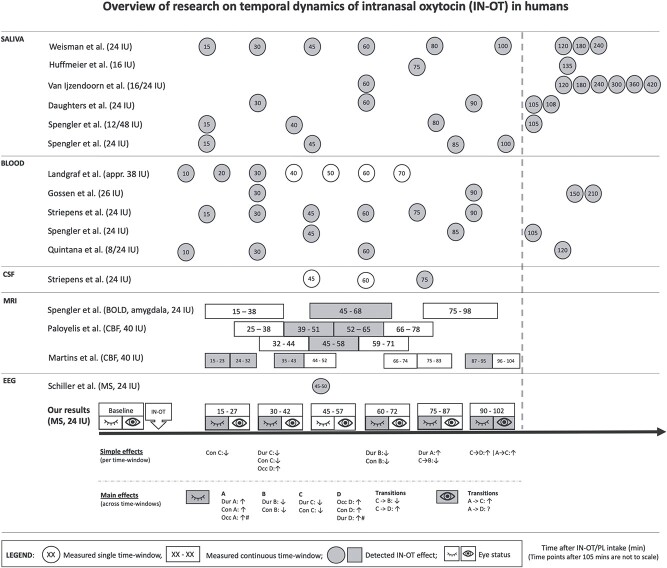
An overview of our experiment’s time frame (time windows with a significant, *P* < 0.05, effect of drug are highlighted in grey, including two borderline-significant trends distinguished with #) in comparison with that of all previous pharmacodynamics studies and the only microstates EEG using IN-OT (time windows with a significant effect of drug are highlighted in grey). Appr. = approximately, BOLD = blood oxygenation level dependent, CBF = cerebral blood flow, CSF = cerebrospinal fluid, EEG = electroencephalography, IN-OT = intranasal oxytocin, IU = international units, MRI = magnetic resonance imaging, MS = microstates, PL = placebo, Dur = duration, Con = contribution, Occ = occurrence, Tran = transition.

We used EEG to characterize the temporal dynamics of IN-OT in humans for the first time, to our knowledge. We tracked IN-OT-induced microstates dynamic changes across the whole male brain cortex (using the dose of 24 IU, most commonly used in neuroscience research, so findings can be of relevance to most past and expected future studies), and across time, during resting-state, from 15 min to 1 h42min (in 6 time windows) post-administration. We found the effects were widespread but diverse, meaning that they were observed on different features of different microstates, between time windows. Next, we first consider a “global” (or any) difference between IN-OT and placebo across time—and compare those results against earlier saliva, blood and MRI pharmacodynamic studies (illustrated in Fig. 3). Second, we discuss the “specific” microstate effects found and their direction and suggest how they can generate or consolidate previous hypotheses for a role of OT in human neurobiology.

### IN-OT Altered Microstate Dynamics across Most of 15 min to 1 h 42min Post-Administration

Overall, we found IN-OT delivery to have functional effects at the resting state brain that started at the same time as reported OT detection in saliva (Huffmeijer et al. 2012; van IJzendoorn et al. 2012; Weisman et al. 2012; Daughters et al. 2015; Spengler et al. 2017), blood plasma (Landgraf 1985; Gossen et al. 2012; Striepens et al. 2013; Quintana et al. 2015; Spengler et al. 2017) (15 min) and the most recent MRI study (as discussed below in detail) (Martins et al. 2020b). This onset time is difficult to compare with that of the (only) CSF study as the latter started measuring OT only at 45 min (and only detected at 75 min—with the limitation of the collection being at the “brain-distant” lumbar region) (Striepens et al. 2013). This effect continued at least until 1 h42min (the end of our recording), with an interruption (45–57 min after IN-OT administration). Thus, although there is some alignment between pharmacodynamics studies across-tissues, in terms of onset, care is advised when inferring OT’s availability, or effect, across tissues (see Fig. 3 for study comparison). In more detail, we found a statistically significant main effect of IN-OT on: contributions of all four canonical microstate classes, duration of microstates A, B, and C, and occurrence of microstate A and D, and transition probabilities (C → B, and C → D), during eyes-closed resting state. During eyes-open, only transition probabilities were significantly different between IN-OT and placebo (A → C and A → D). When looking in time windows separately, our results showed statistically significant effects of IN-OT during eyes-closed in all time windows except time window 3 (45–57 min); and for eyes-open, in time windows 4 (1 h–1 h12 min) and 6 (1 h30 min—1 h42) (see Fig. 3 for a summary). This may suggest a possible decreased (“dip”) effect of IN-OT in the middle time window (45–57 min); however, this was not statistically supported given that no drug by time interactions were statistically significant (further discussed ahead). In terms of main effects of time on microstates dynamics, these were statistically significant, which was expected given the session’s long duration and thus likelihood to induce changes in arousal, drowsiness, etc.—over time. Since our focus was on the effects of drug, we refrained from discussing these results but provide them as supplementary material, so they can used in future research pertaining to the temporal stability of microstates.

### Cognitive Interpretation of IN-OT Effects on Specific Microstates

To procure a hypothesis-generating cognitive meaning for our resting-state microstates findings, and because their employment in neuroscience has been recent, it may be useful to look into resting-state MRI findings—with the caveat that inferences should remain speculative given the still small body of evidence relating both data modalities. Previous studies using both EEG resting-state microstates and MRI resting-state networks, recorded during closed eyes, via spatial correlation (Khanna et al. 2008; Britz et al. 2010; Michel and Koenig 2018) have suggested that: 1) Microstate A is correlated with the auditory and phonological processing network; 2) microstate B with the visual network; and 3) microstate C (albeit by far the least well understood (Michel and Koenig 2018; Krylova et al. 2021)) tentatively with subjective representation of one’s own body (Taylor et al. 2009; Britz et al. 2010), but also with the part of the default mode network linked to self-referential thoughts (Xu et al. 2016) and internally guided cognition (Andrews-Hanna 2012) thus possibly processes directed at one’s self, rather than the external world (Schiller et al. 2019) (but also see (Pipinis et al. 2017) for evidence of a negative link somatic awareness and positive with vigilance levels (Krylova et al. 2021)); and 4) microstate D with the attention network, in particular the reflexive detection of relevant external stimuli, attention-switching and reorientation (Corbetta and Shulman 2002; Britz et al. 2010), and vigilance level (Krylova et al. 2021).

Our data suggest that IN-OT effects on specific microstates may not hold the same profile throughout a 2 h experimental session. Nevertheless, overall (i.e., irrespective of time window), our eyes-closed data, showed IN-OT to induce a greater involvement of microstate A and D, and diminished involvement of microstate B and C (see Fig. 3). The first pattern was reflected in increased duration and greater contribution of microstate A, and longer duration, more frequent occurrence, and larger contribution of D. In light of the microstates and fMRI network findings above-summarized, this would suggest a role of OT in enticing greater resource to language brain resources (linked to A) and to vigilance and external attention-orienting (linked to D)—in order to tune it for the reception of external stimuli, particularly those of the social kind, which are those requiring phonological awareness. As such, our data support both the social saliency (Shamay-Tsoory and Abu-Akel 2016) and the social approach hypotheses of OT (Cohen and Shamay-Tsoory 2018). These cognitive models posit that OT promotes saliency and approach to social stimuli, respectively. Regarding eyes-open, we (only) found increased transition frequency from A to C and to D, suggesting, rather, an IN-OT-provoked *diminished* involvement of A in this eye status, possibly because attention to phonological signs (being mainly auditory in normal-sighted people) is less relevant than in a closed-eyes state (albeit eye-states were not statistically contrasted). The second IN-OT effect pattern we observed, again in eyes-closed, was of a decreased duration and smaller contribution of B and C (and concurrently, a tendency for C to transition more frequent to D, and less to B). This may reflect a lower engagement of visual processing resources (linked to B), which would represent higher resource allocation efficiency given subjects have their eyes closed; and of self-oriented attention (linked to C), but we remind the reader that microstate C is particularly poorly understood.

Notably, our results partially replicate, albeit not at the same time window, the so far single IN-OT microstates study (Schiller et al. 2019). Within the same (and only) time window of Schiller et al.’s (2019) study (45–50 min), we found no effects for either microstate. However, our results’ overall pattern appears to partially agree with that study’s, in that IN-OT also showed: 1) an increased duration of microstate A (albeit they also report it for B, C, and D); 2) a lower contribution of C; and 3) a higher contribution of D. The authors suggested that the observed increased temporal stability (i.e., duration) of microstates reflects the well-known IN-OT anxiolytic effects (Neumann and Slattery 2016) and a shift from internally- to externally-oriented processing modes at rest (as reflected by a decreased presence of microstate C—linked to self-oriented cognition—in favor of external attention-related microstate D), which our data supports. Some differences between studies exist, as Schiller et al. (2019) (Schiller et al. 2019) observed IN-OT to induce lower occurrences of B and C, while we observed lower *contributions* of both, and increased durations of B, C, and D; and we observed higher transition probability from C to A. These disparities may or may not be due to methodological differences: besides using a single time window, that study did not adjust measurements for their pre-administration recording, and used ANOVA; whilst we collected data continuously, and used LMM and baseline correction by entering a pre-administration measurement as a covariate of no interest in our model, specific to *each* session.

Lastly, our recent report of an increased contribution of microstate C and decreased of D in schizophrenia patients (da Cruz et al. 2020) may suggest a promising therapeutic role for IN-OT in the illness, given that we herein find it to have an exactly reverse, thus potentially compensatory, effect. A potential of IN-OT to therapeutically target abnormal microstate dynamics in this disorder is in line with others’ findings (De Berardis et al. 2013; Shilling and Feifel 2016).

### Is There an IN-OT Effect “Dip” at 45–57 min?

Our particular finding of a lack of statistically significant time window-specific (i.e., pair-wise) effects of IN-OT on microstates in the middle time window (45–57 min), both during closed and open eyes, could not be supported by drug by time interactions (as none was statistically significant)—which could have been due to a lack of power in our design. Nevertheless, the possibility of such an effect “interruption or sharp decrease” merits, some discussion, given some previous (and recent) evidence that seems to support it. Although most studies, in a range of different tissues, do not report such a “dip” (Fig. 3), one blood study (Landgraf 1985), the CSF study (Striepens et al. 2013) and, most interestingly, the most recent fMRI study (Martins et al. 2020b) have also not detected OT’s availability/effect in the 45–57 min approximate middle-window of time (with statistically significant drug by time interaction). Using a very similar timeline design (but a higher dose of 40 IU), Martins et al. (2020b) reported an effect at 15–43 min and at 1 h27min—1 h35min after administration, and, also similarly to us, not at 44 min—1 h23 min. This means that the abovementioned IN-OT effect hiatus started in both studies at exactly the same time, and encompassed the 44–45 min time window. (Their effect also lasted longer (49 vs. 18 min in our study), possibly due to the higher dose they used.) A possible explanation, also put forward by those authors, is based on two complementary effects of the IN-OT on the brain. The *earlier* effect of IN-OT (at 15–42 min herein, and 15–43 min in (Martins et al. 2020b)), could be due to the exogenous, inhaled, IN-OT. After the subsequent “dip,” the *later* window of effect (at 1 h–1 h42min herein, and at 1 h27min—1 h35min in (Martins et al. 2020b)) could be due to the “feed forward” release of *endogenous* OT, a mechanism previously suggested (Churchland and Winkielman 2012), whereby administration of the exogenous IN-OT stimulates the release of the endogenous OT (as social-triggered visceral reactions do, via the vagus nerve (Churchland and Winkielman 2012)). Alternatively, but not mutually exclusively, perhaps such “dip” is explained by the inverted U-shape IN-OT concentration-effect model, which posits that when IN-OT accumulates the highest in the synaptic cleft, OT receptor (OTR) activity diminishes temporarily—which could have happened in the middle time window. As previously suggested (Mustoe et al. 2019), a higher IN-OT concentration tends to activate the similarly structured vasopressin receptors, which may reduce OT receptor (OTR) signaling (Borland et al. 2019); or can also lead to OTR desensitization, due to: 1) causing it to couple to different G-protein subtypes (Borland et al. 2019) (leading to desensitization from as early as 5 min of OT stimulation in human embryonic kidney cells (Conti et al. 2009) to 6 h in human myometrium cells (Phaneuf et al. 1998; Robinson et al. 2003)); 2) OTR internalization (Berrada et al. 2000; Conti et al. 2009); 3) OTR mRNA down-regulation (Phaneuf et al. 1997, 1998); or 4) a phosphorylation-dependent mechanism (Gimpl and Fahrenholz 2001; Plested and Bernal 2001).

The only other CBF study (Paloyelis et al. 2016) is more difficult to reconcile with ours and Martins et al.’s (2020b), regarding the “dip” issue. Although those two studies used the same design, OT dose, MRI scanner/site and data modality, they report an opposite progression of the IN-OT effect; i.e., one reports a peak effect of IN-OT in the middle of the observation period (Paloyelis et al. 2016), exactly where the other (and we) reports an interruption (Martins et al. 2020b). This may be because Paloyelis et al. (2016) performed a between-subjects comparison (IN-OT vs. placebo), without accounting for the (existing) pre-administration baseline differences, and made different statistical modeling choices. The fMRI study from Spengler et al. (2017) showed the highest effect of IN-OT (24 IU) in an extended middle window (45 min—1 h 08 min), which overlaps with both our “dip” windows and most of our time window 4 where we also observed an effect. (Martins et al. 2020b) did not report any effect within that extended time window. Nevertheless, our studies are not directly reconcilable with Spengler et al. (2017)‘s task-based fMRI (Spengler et al. 2017) given that their effects were exclusive to high-intensity fear stimuli (with null effects in low-intensity) and only reported on the amygdala (moreover, a region EEG may not fully access). Lastly, even if this “dip” effect of IN-OT is true, it may not affect task-based studies that record over an extended period, as they would have enough power to reach statistical significance when averaging their measurements in each condition, across the task.

### Strengths and Limitations

To examine IN-OT effects on the human brain, we recorded brain activity directly, with high temporal resolution; and used EEG microstates, which allowed an inter-modal bridge with fMRI resting-state networks cognitive proxies (although their sensitivity to sub-cortical function is not yet clear). We excluded potential effects of gender and age, and thus further stratified studies are needed to increase generalizability. We used a semi-automatic pre-processing pipeline validated on resting-state EEG data (da Cruz et al. 2018). LMM statistics allowed us to take into account the pre-administration baseline in each drug session, account for subject-level random effects, and is more appropriate than ANOVA due to the non-independence of microstate data, especially in transition probabilities. We also took into account the amount of IN-OT inhaled.

Limitation-wise, we start with an important disclaimer highlighting that microstates dynamics do not represent all aspects of cortical activity, just as EEG cannot also capture all aspects of brain activity. As such, any absence of findings (in certain time windows) cannot be considered a finding of absence of an effect of IN-OT on brain function as a whole, but rather specifically on microstates dynamics. Second, the data collection was performed in hot weather conditions, which made a few participants impatient. Also, participants’ sweating added noise to our signal in the form of drifts, which may have remained in our data after preprocessing to an extent, which is hard to ascertain. Third, we acknowledge that assuming a single pharmacodynamic model of IN-OT across different brain areas, with a single time course, is overly simplistic. Previous research (Paloyelis et al. 2016; Schiller et al. 2019; Martins et al. 2020b), as well as our own results, suggest a widespread, and time dependent, effect of IN-OT across the brain. Furthermore, as we restricted our study to 1 h42min and we observed IN-OT effects in our last time window we could not locate their end in time. Fourth, we used grand-grand average of both sessions to back-propagate the sorting to individual maps, separately for each time window—instead of averaging across all time windows, which has also been done (Michel and Koenig 2018). Our approach ensured that microstate maps were similar between the sessions at each time window, whilst the latter would have prioritized comparison between time windows—which was not our statistical goal. Spatial correlation between time windows was high, suggesting that our approach was valid. Fifth, we used four microstate classes (A, B, C, and D), which have been previously found to be optimal (Koenig et al. 2002); (Michel and Koenig 2018) and are still the literature standard, although more recent evidence (Custo et al. 2017) suggested a larger number of microstates. We kept the number of microstates at four to ensure better comparability with other microstates studies, in turn allowing us increased interpretability. Sixth, in regard to power, as our data collection took place before any IN-OT’s effects on microstates were published, we could not get reliable effect sizes for an a priori power analysis. However, our sample size is similar to other within-subject and resting state In-OT studies ranging from *N* = 17 for CBF (Martins et al. 2020b) to *N* = 24 for EEG time-frequency analysis (Perry et al. 2010; de Bruijn et al. 2017) which have detected statistically significant effects. Finally, we again highlight the limitation, not regarding methodology but rather data interpretation, that although we have discussed recent literature on inferences relating EEG microstates to fMRI resting state networks—in the hopes it may help interpret our reported IN-OT effects and generate new and better hypotheses for this neuropeptide’s role in cognition—this bridge between modalities should remain speculative given the still small body of evidence associating them.

## Conclusions

We present the first study to our knowledge to examine IN-OT effects on the human brain across a typical neuroscience experimental session time length, using EEG. We observed effects of IN-OT on microstates dynamics from 15 min to 1 h42min post-administration. At the cognitive level, our eyes-closed data suggest IN-OT may heighten at-rest preparatory recruitment of attentional networks tuned for reception of external, and socially relevant stimuli, which, albeit being in line with social salience and approach hypotheses of OT, merits further confirmatory research, given the prior existence of a single microstates IN-OT study. By also providing a comprehensive summary of previous pharmacodynamics IN-OT studies, we hope to help inform study design and interpretability in past and future studies, regarding testing timings and duration, and provide a broader understanding of the role of OT in human cognition.

## Supplementary Material

OTPH_Paper_23_ACCEPTED_supp_mat_bhab404
