# Supplementary material for: Temporal dynamics of intranasal oxytocin in human brain electrophysiology
Source: Cereb Cortex. 2022 Jan 4;32(14):3110–26. doi: 10.1093/cercor/bhab404 (PMC9290557; doi:10.1093/cercor/bhab404)
Supplement: OTPH_Paper_23_ACCEPTED_supp_mat_bhab404 [file otph_paper_23_accepted_supp_mat_bhab404.docx]

## **Supplementary material**


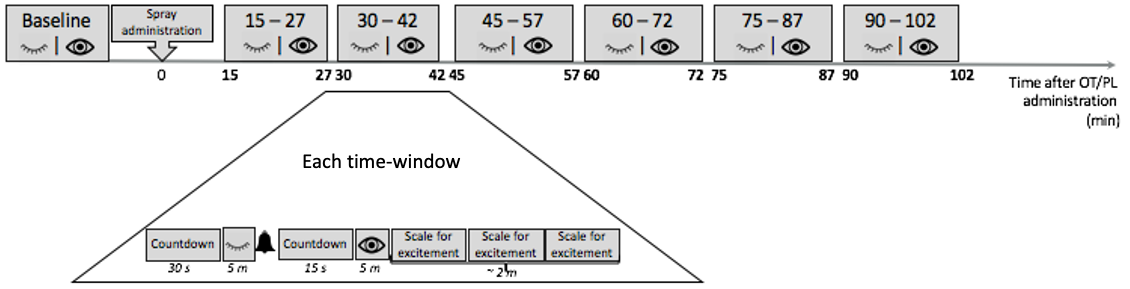


Figure S1 *Experimental design. The study included seven EEG recording time-windows: one prior to the drug administration (baseline) and six post-administration. Each recording time-window started with an on-screen countdown, followed by an on-screen instruction to close the eyes. Eyes-closed recording time was 5 minutes long and ended with an audio signal that instructed the participants to open their eyes. After the signal, there was a 15 seconds long countdown, which was followed by a 5 minutes long eyes open recording. After the eyes open recording, participants were asked to fill on-screen scales for excitement, alertness and sociability (ca. 2 minutes long).*


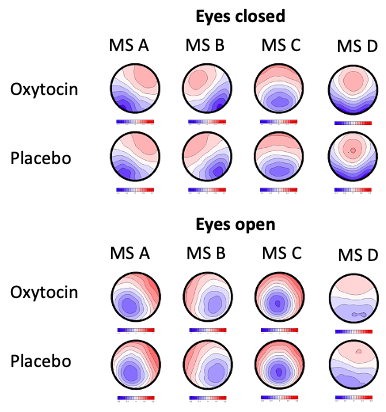


Figure S2 Comparison of each microstate class for IN-OT and PL, for eyes closed (top) and eyes open (bottom). The time-windows were collapsed for visualization purposes. The analysis was performed separately for each time-window and showed that across time-windows and between IN-OT and PL sessions were similar.

## **Supplementary analysis A: Regression analysis of oxytocin bottle weight**

To assess the effect of the IN-OT amount (measured as the subtraction of the post-administration by the pre-administration bottle weights) in our analysis, we performed a series of linear regressions with the independent variables: IN-OT amount, baseline values of microstate feature, and time; dependent variable: microstate feature. The regression coefficient analysis showed statistical significance (<.05) of the IN-OT amount coefficient – meaning the IN-OT amount had a significant effect on the microstate feature - in only two out of 24 regressions on eyes closed data and only four out of 24 regressions in eyes open data (results of this analysis, along with bottle weight data, are reported in the tables below). Since we found the IN-OT amount to be a significant predictor in a very small fraction of the microstates features, we performed all future analysis without accounting for the inhaled IN-OT amount.

### **Eyes closed**

Table A-1. Regression analysis of oxytocin bottle weight, eyes closed. Significant values (p<.05) are marked with an asterisk (*).

| Microstate feature | Correlation –  Bottle weight vs. microstate feature,  Pearson | Correlation –  Bottle weight vs. microstate feature  significance | Significance of coefficients – bottle weight |
| --- | --- | --- | --- |
| Duration ms A | .092 | .164 | .994 |
| Duration ms B | .030 | .337 | .418 |
| Duration ms C | .004 | .484 | .228 |
| Duration ms D | .112 | .117 | .660 |
| Occurrence ms A | -.036 | .353 | .151 |
| Occurrence ms B | -.183 | .026 | .795 |
| Occurrence ms C | -.052 | .293 | .700 |
| Occurrence ms D | -.025 | .395 | .868 |
| Contribution ms A | .019 | .420 | .274 |
| Contribution ms B | -.092 | .165 | .984 |
| Contribution ms C | -.016 | .436 | .498 |
| Contribution ms D | .079 | .203 | .772 |
| Transition ms A→B | -.115 | .111 | .234 |
| Transition ms A→C | -.155 | .050 | .217 |
| Transition ms A→D | .171 | .035* | .036* |
| Transition ms B→A | .105 | .133 | .100 |
| Transition ms B→C | -.120 | .102 | .860 |
| Transition ms B→D | .053 | .287 | .545 |
| Transition ms C→A | -220 | .009* | .014* |
| Transition ms C→B | .057 | .272 | .238 |
| Transition ms C→D | .094 | .161 | .724 |
| Transition ms D→A | .050 | .299 | .293 |
| Transition ms D→B | -.142 | .066 | .671 |
| Transition ms D→C | .007 | .472 | .496 |

### **Eyes open**

Table A-2. Regression analysis of oxytocin bottle weight, eyes open. Significant values (p<.05) are marked with an asterisk (*).

| Microstate feature | Correlation –  Bottle weight vs. microstate feature,  Pearson | Correlation –  Bottle weight vs. microstate feature  significance | Significance of coefficients – bottle weight |
| --- | --- | --- | --- |
| Duration_ms A | -.143 | .065 | .085 |
| Duration ms B | -.193 | .020 | .119 |
| Duration ms C | .035 | .355 | .177 |
| Duration ms D | -.354 | <.001* | <.001* |
| Occurrence ms A | .131 | .082 | .015* |
| Occurrence ms B | .035 | .352 | .014* |
| Occurrence ms C | .252 | .003* | .216 |
| Occurrence_ms D | .020 | .416 | .083 |
| Contribution ms A | .006 | .474 | .389 |
| Contribution ms B | -.079 | .201 | .409 |
| Contribution ms C | .192 | .021* | .707 |
| Contribution ms D | -.149 | .057 | .429 |
| Transition ms A→B | -.114 | .113 | .692 |
| Transition ms A→C | .083 | .191 | .938 |
| Transition ms A→D | .004 | .485 | .927 |
| Transition ms B→A | .072 | .223 | .570 |
| Transition ms B→C | .192 | .021* | .047* |
| Transition ms B→D | -.211 | .012* | .209 |
| Transition ms C→A | -.029 | .380 | .614 |
| Transition ms C→B | .099 | .148 | .677 |
| Transition ms C→D | -.029 | .381 | .768 |
| Transition ms D→A | -.076 | .212 | .775 |
| Transition ms D→B | -.092 | .166 | .577 |
| Transition ms D→C | .180 | .027* | .801 |

### **Bottle weight data**

Table A-3. Weight of nasal spray bottles before and after drug inhalation, and the amount of drug inhaled.

|  | OT | | | PL | | |
| --- | --- | --- | --- | --- | --- | --- |
| Participant ID | Weight of spray bottle before administration | Weight of spray bottle before administration | Amount of drug inhaled | Weight of spray bottle before administration | Weight of spray bottle before administration | Amount of drug inhaled |
| 01 | 37.55 | 36.79 | 0.76 | 37.8 | 37.05 | 0.75 |
| 02 | 37.68 | 37 | 0.68 | Missing data | | Averaged value – 0.69 |
| 03 | 37.6624 | 37.1413 | 0.5211 | 37.9333 | 36.5191 | 1.4142 |
| 04 | 36.79 | 36.07 | 0.72 | 37.06 | 36.64 | 0.42 |
| 05 | Participant excluded due to recording difficulties | | | | | |
| 06 | 37.96 | 37.32 | 0.64 | 37.8173 | 37.2229 | 0.5944 |
| 07 | 37.578 | 36.8149 | 0.7631 | 37.8087 | 37.1765 | 0.6322 |
| 08 | 36.207 | 35.906 | 0.301 | 37.1862 | 36.7761 | 0.4101 |
| 09 | 37.5731 | 36.8003 | 0.7728 | 37.9338 | 37.2005 | 0.7333 |
| 10 | 36.8102 | 36.0234 | 0.7868 | 37.208 | 36.3732 | 0.8348 |
| 11 | 37.7451 | 37.0311 | 0.714 | 37.9434 | 37.3304 | 0.613 |
| 12 | 37.0417 | 36.2228 | 0.8189 | 37.3432 | 36.6389 | 0.7043 |
| 13 | 37.8112 | 37.152 | 0.6592 | 37.6448 | 37.1074 | 0.5374 |
| 14 | 37.1361 | 36.321 | 0.8152 | 38.0649 | 37.4323 | 0.6326 |
| 15 | 37.5992 | 36.8805 | 0.7187 | 37.8266 | 37.1095 | 0.7171 |
| 16 | 36.8965 | 36.4024 | 0.4941 | 37.1161 | 36.4853 | 0.6308 |
| 17 | 37.7266 | 37.1431 | 0.5835 | 37.8115 | 37.2924 | 0.5191 |
| 18 | 37.1431 | 36.4325 | 0.7106 | 37.2924 | 36.4853 | 0.8071 |
| 19 | 37.7006 | 36.8527 | 0.8479 | 37.8379 | 37.1185 | 0.7194 |
| 20 | 36.8567 | 36.2518 | 0.6049 | 37.1284 | 36.3369 | 0.7915 |

**Supplementary material B – topographic analysis of microstate maps**

Table B-1. TANOVA p-values. (Oxytocin vs placebo). TP = time-window, MS = microstate

1. Eyes closed

|  | TP 0 | TP 1 | TP 2 | TP 3 | TP 4 | TP 5 | TP6 |
| --- | --- | --- | --- | --- | --- | --- | --- |
| MS A | 0,3184 | 0,0004 | 0,0018 | 0,3192 | 0,0356 | 0,0036 | 0,5384 |
| MS B | 0,3324 | 0,0028 | 0,0006 | 0,8694 | 0,007 | 0,7236 | 0,0026 |
| MS C | 0,0196 | 0,0002 | 0,0112 | 0,4098 | 0,099 | 0,0006 | 0,327 |
| MS D | 0,5364 | 0,2238 | 0,0002 | 0,6188 | 0,0296 | 0,004 | 0,1718 |

1. Eyes open

|  | TP 0 | TP 1 | TP 2 | TP 3 | TP 4 | TP 5 | TP 6 |
| --- | --- | --- | --- | --- | --- | --- | --- |
| MS A | 0,0122 | 0,1072 | 0,284 | 0,6448 | 0,0958 | 0,2688 | 0,1822 |
| MS B | 0,1816 | 0,5584 | 0,7202 | 0,5512 | 0,6134 | 0,5008 | 0,8732 |
| MS C | 0,1466 | 0,4488 | 0,5084 | 0,0922 | 0,6358 | 0,3272 | 0,7556 |
| MS D | 0,0016 | 0,1672 | 0,4202 | 0,5318 | 0,5132 | 0,4958 | 0,1616 |

Table B-2. Spatial correlation coefficients between microstate class topographies in the IN-OT and the PL conditions. TP = time-window, MS = microstate

1. Eyes closed

|  | TP 0 | TP 1 | TP 2 | TP 3 | TP 4 | TP 5 | TP 6 |
| --- | --- | --- | --- | --- | --- | --- | --- |
| MS A | 0,990857 | 0,900644 | 0,955519 | 0,981321 | 0,962226 | 0,921263 | 0,991281 |
| MS B | 0,982243 | 0,919163 | 0,909933 | 0,993789 | 0,933583 | 0,997248 | 0,91759 |
| MS C | 0,978605 | 0,896714 | 0,952842 | 0,983909 | 0,967654 | 0,937145 | 0,983468 |
| MS D | 0,993528 | 0,987738 | 0,928072 | 0,99378 | 0,951525 | 0,954955 | 0,981071 |

1. Eyes open

|  | TP 0 | TP 1 | TP 2 | TP 3 | TP 4 | TP 5 | TP 6 |
| --- | --- | --- | --- | --- | --- | --- | --- |
| MS A | 0,949154 | 0,428849 | 0,981475 | 0,846654 | 0,763222 | 0,978559 | 0,655003 |
| MS B | 0,952647 | 0,724668 | 0,995748 | 0,771952 | 0,549442 | 0,9649 | 0,979322 |
| MS C | 0,974245 | 0,715775 | 0,987877 | 0,631209 | 0,798543 | 0,980334 | 0,877666 |
| MS D | 0,918254 | 0,744638 | 0,991601 | 0,753441 | 0,832574 | 0,991867 | 0,471749 |

Table B-3. Dissimilarity indices between microstate topographies in the IN-OT and the PL conditions. TP = time-window, MS = microstate

1. Eyes closed

|  | TP 0 | TP 1 | TP 2 | TP 3 | TP 4 | TP 5 | TP 6 |
| --- | --- | --- | --- | --- | --- | --- | --- |
| MS A | 0,135224 | 0,445771 | 0,298264 | 0,193283 | 0,27486 | 0,39683 | 0,132054 |
| MS B | 0,18845 | 0,402086 | 0,424422 | 0,111451 | 0,364464 | 0,074187 | 0,405979 |
| MS C | 0,206858 | 0,454501 | 0,307109 | 0,179391 | 0,254348 | 0,354555 | 0,181833 |
| MS D | 0,113769 | 0,156603 | 0,379283 | 0,111532 | 0,311369 | 0,300149 | 0,194571 |

1. Eyes open

|  | TP 0 | TP 1 | TP 2 | TP 3 | TP 4 | TP 5 | TP 6 |
| --- | --- | --- | --- | --- | --- | --- | --- |
| MS A | 0,318893 | 1,068785 | 0,192485 | 0,553799 | 0,688154 | 0,20708 | 0,830658 |
| MS B | 0,307744 | 0,742068 | 0,092213 | 0,675349 | 0,949271 | 0,264951 | 0,203364 |
| MS C | 0,226956 | 0,753956 | 0,155714 | 0,858827 | 0,634756 | 0,198323 | 0,494639 |
| MS D | 0,404342 | 0,71465 | 0,129611 | 0,702223 | 0,578663 | 0,127542 | 1,027863 |

## **Supplementary material C: Statistical measures of microstate features extracted from eyes closed and eyes open data**

The LMM model and the subsequent tests were implemented in R, using *lmer* package (55)l, as follows:

*m = lmer(X ~ BASE + T*DRUG + (1|id).*

*X =microstate feature or transition probability value*

*BASE = value of X in the pre-administration time-window*

*T*DRUG = interaction between time-window and placebo/oxytocin*

The F-statistic and associated p-values were derived using *anova* function:

*anova(m, type=III)*.

### **Eyes closed**

Table C-1. Summary of statistical analysis of microstate features of eyes closed data. Significant values (p<.05) are marked with an asterisk (*). Trends (p<.10) are marked with a double asterisk (**)

| **Microstate feature** | **Variable** | **Statistic measures** |
| --- | --- | --- |
| **MICROSTATE A** | | |
| Duration | Base  Time-window  Drug  Time*Drug | \| F(1, 35.6) = 23.57, p < 0.001* \| \| --- \| \| F(5, 188.3) = 31.14, p < 0.001* \| \| F(1, 190.4) = 5.05, p = 0.026* \| \| F(5, 188.4) = 0.67, p = 0.644 \| |
| Occurrence | Base  Time-window  Drug  Time*Drug | \| F(1, 130) = 107.64, p < 0.001* \| \| --- \| \| F(5, 188) = 28.16, p < 0.001* \| \| F(1, 194) = 3.83, p = 0.052** \| \| F(5, 188) = 1.15, p = 0.336 \| |
| Contribution | Base  Time-window  Drug  Time*Drug | \| F(1, 143) = 75.27, p < 0.001* \| \| --- \| \| F(5, 187) = 45.44, p < 0.001* \| \| F(1, 190) = 8.57, p = 0.004* \| \| F(5, 187) = 0.17, p = 0.973 \| |
| **MICROSTATE B** | | |
| Duration | Base  Time-window  Drug  Time*Drug | \| F(1, 35.5) = 94.96, p < 0.001* \| \| --- \| \| F(5, 188.2) = 22.58, p < 0.001* \| \| F(1, 194.3) = 5.82, p = 0.017* \| \| F(5, 188.2) = 0.9, p = 0.479 \| |
| Occurrence | Base  Time-window  Drug  Time*Drug | \| F(1, 31.4) = 128.5, p < 0.001* \| \| --- \| \| F(5, 188.6) = 26.42, p < 0.001* \| \| F(1, 194.5) = 1.66, p = 0.2 \| \| F(5, 188.6) = 1, p = 0.42 \| |
| Contribution | Base  Time-window  Drug  Time*Drug | \| F(1, 35.4) = 136.9, p < 0.001* \| \| --- \| \| F(5, 188.7) = 41.3, p < 0.001* \| \| F(1, 193.5) = 10, p = 0.002* \| \| F(5, 188.7) = 0.8, p = 0.549 \| |
| **MICROSTATE C** | | |
| Duration | Base  Time-window  Drug  Time*Drug | \| F(1, 177) = 5.87, p = 0.016* \| \| --- \| \| F(5, 187) = 40.1, p < 0.001* \| \| F(1, 188) = 6.68, p = 0.011* \| \| F(5, 187) = 1.94, p = 0.09** \| |
| Occurrence | Base  Time-window  Drug  Time*Drug | \| F(1, 41.2) = 36.39, p < 0.001* \| \| --- \| \| F(5, 188.9) = 69.9, p < 0.001* \| \| F(1, 194.8) = 2.26, p = 0.13 \| \| F(5, 188.9) = 0.69, p = 0.63 \| |
| Contribution | Base  Time-window  Drug  Time*Drug | \| F(1, 162) = 13.54, p < 0.001* \| \| --- \| \| F(5, 188) = 125.36, p < 0.001* \| \| F(1, 189) = 10.59, p = 0.001* \| \| F(5, 188) = 0.83, p = 0.53 \| \|  \| |
| **MICROSTATE D** | | |
| Duration | Base  Time-window  Drug  Time*Drug | \| F(1, 47.8) = 52.24, p <0.001* \| \| --- \| \| F(5, 189) = 7.95, p < 0.001* \| \| \| F(1, 191.3) = 3.6, p = 0.059** \| \| \| \| F(5, 189) = 0.36, p = 0.872 \| |
| Occurrence | Base  Time-window  Drug  Time*Drug | \| F(1, 141) = 38.7, p < 0.001* \| \| --- \| \| F(5, 184) = 8.27, p < 0.001* \| \| F(1, 186) = 4.36, p = 0.038* \| \| F(5, 184) = 1.21, p = 0.304 \| |
| Contribution | Base  Time-window  Drug  Time*Drug | \| F(1, 87.4) = 44.39, p < 0.001* \| \| --- \| \| F(5, 188.9) = 3.44, p = 0.005* \| \| F(1, 191.2) = 5.65, p = 0.019* \| \| F(5, 188.9) = 0.25, p = 0.940 \| |
| **TRANSITIONS** | | |
| A🡪B | Base  Time-window  Drug  Time*Drug | \| F(1, 17,665) = 19,53, p < 0.001* \| \| --- \| \| F(5, 181,052) = 1,01, p = 0,413 \| \| F(1, 191,435) = 1,37, p = 0,243 \| \| F(5, 181,104) = 0,30, p = 0,911 \| |
| A🡪C | Base  Time-window  Drug  Time*Drug | \| F(1, 97,165) = 4,99, p = 0,028* \| \| --- \| \| F(5, 185,047) = 3,64, p = 0,004* \| \| F(1, 200,351) = 0,69, p = 0,407 \| \| F(5, 185,066) = 0,51, p = 0,765 \| |
| A🡪D | Base  Time-window  Drug  Time*Drug | \| F(1, 41,294) = 10,41, p = 0,002* \| \| --- \| \| F(5, 188,774) = 5,93, p < 0.001* \| \| F(1, 205,303) = 1,96, p = 0,163 \| \| F(5, 188,788) = 1,58, p = 0,167 \| |
| B🡪A | Base  Time-window  Drug  Time*Drug | \| F(1, 76,409) = 38,91, p < 0.001* \| \| --- \| \| F(5, 186,225) = 0,93, p = 0,461 \| \| F(1, 198,696) = 0,01, p = 0,938 \| \| F(5, 186,273) = 0,84, p = 0,523 \| |
| B🡪C | Base  Time-window  Drug  Time*Drug | \| F(1, 49,058) = 8,89, p = 0,004* \| \| --- \| \| F(5, 171,239) = 1,93, p = 0,092** \| \| F(1, 175,167) = 2,32, p = 0,129 \| \| F(5, 171,263) = 1,18, p = 0,319 \| |
| B🡪D | Base  Time-window  Drug  Time*Drug | \| F(1, 154,01) = 9,73, p = 0,002* \| \| --- \| \| F(5, 185,75) = 4,7, p < 0.001* \| \| F(1, 193,48) = 3,26, p = 0,072** \| \| F(5, 185,78) = 0,36, p = 0,877 \| |
| C🡪A | Base  Time-window  Drug  Time*Drug | \| F(1, 62,268) = 7,56, p = 0,008* \| \| --- \| \| F(5, 188,604) = 2,37, p = 0,041* \| \| F(1, 194,4) = 0,52, p = 0,473 \| \| F(5, 188,644) = 1,77, p = 0,12 \| |
| C🡪B | Base  Time-window  Drug  Time*Drug | \| F(1, 179,18) = 2,37, p = 0,125 \| \| --- \| \| F(5, 186,43) = 2,1255, p = 0,064** \| \| F(1, 189,85) = 4,5762, p = 0,034* \| \| F(5, 186,45) = 0,9014, p = 0,481 \| |
| C🡪D | Base  Time-window  Drug  Time*Drug | \| F(1, 90,887) = 5,97, p = 0,016* \| \| --- \| \| F(5, 188,326) = 2,64, p = 0,025* \| \| F(1, 191,18) = 4,06, p = 0,045* \| \| F(5, 188,346) = 1,64, p = 0,151 \| |
| D🡪A | Base  Time-window  Drug  Time*Drug | \| F(1, 46,371) = 38,00, p < 0.001* \| \| --- \| \| F(5, 188,683) = 1,49, p = 0,196 \| \| F(1, 191,4) = 1,91, p = 0,168 \| \| F(5, 188,699) = 0,85, p = 0,514 \| |
| D🡪B | Base  Time-window  Drug  Time*Drug | F(1, 21,859) = 148,19, p < 0.001*  F(5, 190,032) = 2,17, p = 0,059**  F(1, 196,27) = 2,06, p = 0,152  F(5, 190,106) = 0,42, p = 0,832 |
| D🡪C | Base  Time-window  Drug  Time*Drug | \| F(1, 107,83) = 10,26, p = 0,002* \| \| --- \| \| F(5, 188,78) = 7,18, p < 0.001* \| \| F(1, 192,67) = 0,17, p = 0,678 \| \| F(5, 188,81) = 0,20, p = 0,964 \| |

### **Eyes open**

Table C-2. Summary of statistical analysis of microstate features of eyes open data. Significant values (p<.05) are marked with an asterisk (*). Trends (p<.10) are marked with a double asterisk (**)

| **Microstate feature** | **Variable** | **Statistic measures** |
| --- | --- | --- |
| **MICROSTATE A** | | |
| Duration | Base  Time-window  Drug  Time*Drug | \| F(1, 60.867) = 26.40, p <0.001* \| \| --- \| \| F(5, 181.451) = 11.52, p <0.001* \| \| F(1, 204.886) = 1.51, p = 0.22 \| \| F(5, 181.463) = 0.76, p = 0.578 \| |
| Occurrence | Base  Time-window  Drug  Time*Drug | \| F(1, 107.09) = 61.51, p <0.001* \| \| --- \| \| F(5, 187.54) = 14.65, p <0.001* \| \| F(1, 189.95) = 2.29, p = 0.132 \| \| F(5, 187.53) = 0.43, p = 0.827 \| |
| Contribution | Base  Time-window  Drug  Time*Drug | \| F(1, 47.105) = 83.39, p <0.001* \| \| --- \| \| F(5, 188.091) = 21.71, p <0.001* \| \| F(1, 196.781) = 0.03, p = 0.859  F(5, 188.091) = 0.42, p = 0.834 \| |
| **MICROSTATE B** | | |
| Duration | Base  Time-window  Drug  Time*Drug | \| F(1, 196.62) = 4.22, p = 0.041* \| \| --- \| \| F(5, 183.89) = 8.51, p <0.001* \| \| F(1, 185.18) = 0.78, p = 0.378 \| \| F(5, 183.89) = 1.43, p = 0.214 \| |
| Occurrence | Base  Time-window  Drug  Time*Drug | \| F(1, 115.02) = 9.17, p = 0.003* \| \| --- \| \| F(5, 177.21) = 16.2, p <0.001* \| \| F(1, 191.9) = 0.21, p = 0.647 \| \| F(5, 177.22) = 0.69, p = 0.635 \| |
| Contribution | Base  Time-window  Drug  Time*Drug | \| F(1, 128.13) = 11.27, p = 0.001* \| \| --- \| \| F(5, 179.78) = 17.07, p <0.001* \| \| F(1, 187.08) = 0.79, p = 0.374 \| \| F(5, 179.78) = 0.59, p = 0.708 \| |
| **MICROSTATE C** | | |
| Duration | Base  Time-window  Drug  Time*Drug | \| F(1, 115.1) = 22.61, p <0.001* \| \| --- \| \| F(5, 184.05) = 5.75, p <0.001* \| \| F(1, 199.61) = 2.02, p = 0.157 \| \| F(5, 184.03) = 0.86, p = 0.51 \| |
| Occurrence | Base  Time-window  Drug  Time*Drug | \| F(1, 157.35) = 31.80, p <0.001* \| \| --- \| \| F(5, 181.96) = 25.62, p <0.001* \| \| F(1, 184.82) = 0.47, p = 0.493 \| \| F(5, 181.97) = 0.83, p = 0.528 \| |
| Contribution | Base  Time-window  Drug  Time*Drug | \| F(1, 78.706) = 67.89, p <0.001* \| \| --- \| \| F(5, 186.124) = 21.18, p<0.001* \| \| F(1, 191.338) = 3.02, p = 0.084** \| \| F(5, 186.142) = 0.87, p = 0.503 \| |
| **MICROSTATE D** | | |
| Duration | Base  Time-window  Drug  Time*Drug | \| F(1, 160.51) = 10.67, p = 0.001* \| \| --- \| \| F(5, 189.46) = 3.01, p = 0.012* \| \| F(1, 201.1) = 0.01, p = 0.904 \| \| F(5, 189.46) = 0.83, p = 0.528 \| |
| Occurrence | Base  Time-window  Drug  Time*Drug | \| F(1, 95.205) = 21.98, p <0.001* \| \| --- \| \| F(5, 191.153) = 2.37, p = 0.041* \| \| F(1, 200.647) = 3.08, p = 0.08** \| \| F(5, 191.153) = 0.69, p = 0.628 \| |
| Contribution | Base  Time-window  Drug  Time*Drug | \| F(1, 93.199) = 25.68, p <0.001* \| \| --- \| \| F(5, 190.71) = 3.30, p = 0.007* \| \| F(1, 205.766) = 2.31, p = 0.130 \| \| F(5, 190.71) = 0.85, p = 0.515 \| |
| **TRANSITIONS** | | |
| A🡪B | Base  Time-window  Drug  Time*Drug | \| F(1, 33,155) = 1,3079, p = 0,261 \| \| --- \| \| F(5, 187,208) = 1,8734, p = 0,101 \| \| F(1, 194,327) = 0,0547, p = 0,815 \| \| F(5, 187,217) = 1,5073, p = 0,189 \| |
| A🡪C | Base  Time-window  Drug  Time*Drug | \| F(1, 67,724) = 0,7267, p = 0,397 \| \| --- \| \| F(5, 187,983) = 6,0348, p <0.001* \| \| F(1, 202,265) = 0,6492, p = 0,421 \| \| F(5, 188,001) = 2,9504, p = 0,013* \| |
| A🡪D | Base  Time-window  Drug  Time*Drug | \| F(1, 31,211) = 0,0006, p = 0,981 \| \| --- \| \| F(5, 187,758) = 3,0222, p = 0,012* \| \| F(1, 204,901) = 0,0137, p = 0,907 \| \| F(5, 187,756) = 2,3019, p = 0,046* \| |
| B🡪A | Base  Time-window  Drug  Time*Drug | \| F(1, 111,45) = 0,7652, p = 0,384 \| \| --- \| \| F(5, 187,15) = 4,215, p = 0,001* \| \| F(1, 196,66) = 0,5093, p = 0,476 \| \| F(5, 187,16) = 0,915, p = 0,472 \| |
| B🡪C | Base  Time-window  Drug  Time*Drug | \| F(1, 55,404) = 0,0469, p = 0,829 \| \| --- \| \| F(5, 187,067) = 8,1416, p <0.001* \| \| F(1, 190,237) = 0,7776, p = 0,379 \| \| F(5, 187,065) = 0,3365, p = 0,890 \| |
| B🡪D | Base  Time-window  Drug  Time*Drug | \| F(1, 189,95) = 0,3461, p = 0,557 \| \| --- \| \| F(5, 186,25) = 8,1681, p <0.001* \| \| F(1, 191,43) = 0,3244, p = 0,57 \| \| F(5, 186,26) = 2,1699, p = 0,059** \| |
| C🡪A | Base  Time-window  Drug  Time*Drug | \| F(1, 52,813) = 0,2862, p = 0,595 \| \| --- \| \| F(5, 188,674) = 2,1652, p = 0,060** \| \| F(1, 194,284) = 3,3642, p = 0,068** \| \| F(5, 188,668) = 0,8372, p = 0,525 \| |
| C🡪B | Base  Time-window  Drug  Time*Drug | \| F(1, 101,56) = 4,6031, p = 0,034* \| \| --- \| \| F(5, 185,85) = 3,001, p = 0,012* \| \| F(1, 191,8) = 2,4761, p = 0,117 \| \| F(5, 185,86) = 0,6313, p = 0,676 \| |
| C🡪D | Base  Time-window  Drug  Time*Drug | \| F(1, 65,081) = 0,8568, p = 0,358 \| \| --- \| \| F(5, 183,208) = 3,5682, p = 0,004* \| \| F(1, 187,272) = 1,3253, p = 0,251 \| \| F(5, 183,216) = 0,6044, p = 0,697 \| |
| D🡪A | Base  Time-window  Drug  Time*Drug | \| F(1, 31,713) = 4,1344, p = 0,050** \| \| --- \| \| F(5, 187,111) = 1,7124, p = 0,134 \| \| F(1, 191,037) = 0,0076, p = 0,931 \| \| F(5, 187,123) = 0,5426, p = 0,744 \| |
| D🡪B | Base  Time-window  Drug  Time*Drug | \| F(1, 31,019) = 7,2936, p = 0,011* \| \| --- \| \| F(5, 187,311) = 2,1012, p = 0,067** \| \| F(1, 194,647) = 0,081, p = 0,776 \| \| F(5, 187,316) = 1,2029, p = 0,309 \| |
| D🡪C | Base  Time-window  Drug  Time*Drug | \| F(1, 110,64) = 0,8369, p = 0,362 \| \| --- \| \| F(5, 187,18) = 2,454, p = 0,035* \| \| F(1, 191,03) = 0,6082, p = 0,436 \| \| F(5, 187,17) = 0,2375, p = 0,946 \| |
